# Nutritional efficiency of feed restricted F1 Holstein/Zebu cows during the middle third of lactation

**DOI:** 10.5713/ajas.18.0795

**Published:** 2019-05-28

**Authors:** Pedro Felipe Santana, Vicente Ribeiro Rocha Júnior, José Reinaldo Mendes Ruas, Flávio Pinto Monção, Luana Alcântara Borges, Thaís Eleonora Santos Sousa, Fredson Vieira e Silva, Walber de Oliveira Rabelo, Cinara da Cunha Siqueira Carvalho, Eleuza Clarete Junqueira de Sales

**Affiliations:** 1 Department of Animal Science and Technology, State University of Montes Claros, Avenue Reinaldo Viana, Janaúba, MG 2630, Brazil; 2 National Institute of Science and Technology –INCT Animal Science Member, Brasília, 71605-001, Brazil

**Keywords:** Dairy Cattle, Feeding Time, Milk Production, Nitrogen Balance, Plasma Urea Nitrogen

## Abstract

**Objective:**

The objective of this study was to evaluate the effects of different levels of quantitative feed restriction on nutrient intake and digestibility, nitrogen balance, efficiency and feeding behavior, and productive performance in F1 Holstein/Zebu cows during the middle third of their lactation.

**Methods:**

Sixty F1 Holstein/Zebu cows with 111.5±11.75 days of lactation and an initial body weight (BW) of 499±30 kg (mean±standard error of the mean) were used. The experimental design was completely randomized with the following diet levels of feed restriction: 3.39%, 2.75%, 2.50%, 2.25%, and 2.00% of BW, with 12 replications for each level. The experiment lasted for 63 days, of which each period lasted 21 days with the first 16 days for diet adaptation followed by 5 days for collection of data and samples.

**Results:**

For each 1% of BW diet restriction, there was a decrease in dry matter intake of 5.26 kg/d (p<0.01). There was no difference in daily milk production (p = 0.09) under the restriction levels of 3.39% to 2.0% of BW. When corrected for 3.5% fat, milk production declined (p = 0.05) 3.46 kg/d for each percentage unit of feed restriction.

**Conclusion:**

Restricting the feed supply for F1 Holstein/Zebu cows in the middle third of their lactation period altered nutrient intake, nitrogen balance and ingestive behavior but did not affect milk production or feed efficiency. However, considering the observed BW loss and decrease in milk production corrected for 3.5% fat, restriction of no less than 2.5% BW is recommended.

## INTRODUCTION

The dairy industry has a substantial contribution to the Brazilian agribusiness and most of the milk that is produced is from crossbred Holstein/Zebu cows [1]. These animals are used because of their consistent ability to maintain high levels of performance, with no changes in management, as well as their rusticity and adaptability to changes that occur in tropical environments [2].

Milk production (MP) in Brazil has nutritional value, and the use of forage plants whose supply and nutritional value changes considerably throughout the year, mainly during the dry season, affecting the animals’ performance [3]. One alternative used by producers to maintain MP and to ensure its permanence is the strategic use of feedlots for animals whose food costs can have a negative impact on production.

Feed restriction may be an alternative that reduces production costs and increases the feed efficiency of the cattle, as reported by Keogh et al [4]. However, there is still little knowledge regarding the effects of feed restriction on nutritional, productive and behavioral parameters of crossbred Holstein/Zebu cows during the middle third of the lactation period in the literature. According to Schütz et al [5], restriction of dietary supply in lactating cows can trigger rapid mobilization of body tissues for maintenance of normal bodily functions and can also affect the productive and reproductive activities of the animal, causing adaptive and metabolic body changes. There may also be a reduction in MP [6] as well as in fat, protein and stability [7,8] beginning the fifth day of restriction. There may also be an increase in the somatic cell count [9]. However, the results may vary depending on the severity, duration and type of feed restriction [6]. In addition to the lactation phase, it is important to study the best level of this restriction for the animals and to relate these results to production [2].

The objective of this study was to evaluate the effects of dif ferent levels of quantitative food restriction on the nutrient intake and digestibility, nitrogen balance, MP, feed efficiency, and ingestive behavior in F1 Holstein/Zebu cows during the middle third of the lactation period.

## MATERIALS AND METHODS

### Animal care and location

All procedures involving animals was approved by the institutional committee on animal use (protocol number 138/2017). The study was conducted at the Experimental Farm of Unimontes, GPS coordinates: latitude 15°52′38″ S, longitude 43°20′05″ W.

### Animals, experimental design, diet and management

Sixty F1 Holstein/Zebu cows in the middle third of their lactation periods (111.5±11.75 days of lactation) with an initial body weight (BW) of 499±30 kg (mean±standard error of the mean) and a mean age of 6 years were used. The experimental design was completely randomized and had five feed restriction levels (3.39%, 2.75%, 2.50%, 2.25%, 2.00%) with twelve cows used for each treatment. The diet supply, kg dry matter (DM)/d, defined as percentage of BW, were: *ad libitum* (3.39%), allowing 5% of refusals relative to the amount of DM provided, and diets provided with 2.75%, 2.50%, 2.25%, and 2.00% of BW. Before the trial period all cows received the experimental diet provided *ad libitum* for 14 days, with a goal of allowing the animals to adapt to the diet and management.

The diet (Table 1) was given based on the BW of each cow and in accordance with each treatment, maintaining roughage: concentrate ratio of 75:25 in the total DM of the diet. The diets were offered to the animals twice a day, at 8:00 am and at 3:00 pm, in a complete diet system. The roughage base for the diets was corn silage, which was weighed daily and then mixed into the concentrate.

The cows were kept in individual pens with an area of ap proximately 26 m^2^, equipped with troughs (1 linear meter) and drinkers (capacity of 200 liters). Milking was performed mechanically twice a day, at 7:00 am and 2:00 pm, with the calf present to stimulate milk letdown. Immediately after milking, the calves remained with the cows to feed from the residual milk.

The experiment lasted for 63 days that were divided in three period. Each period lasted 21 days with the first 16 days for adaptation of the animals the diet (diet supply levels) followed by 5 days for collection of data and samples.

### Intake and apparent digestibility evaluations

Intake was evaluated daily by weighing the feed provided and the refusals of the animals. Samples of the offered diets and the refusals were stored at −20°C for further analysis.

Samples of diets, concentrate ingredients, refusals, and feces were analyzed to evaluate feed intake and digestibility. The samples were analyzed for DM (method 967.03), ash (method 942.05), crude protein (CP; method 981.10), and ether extract (EE; method 920.39) according to the recommendations of the AOAC [10]. The contents of the neutral detergent fiber were corrected for ash and protein (NDFap) using heat-stable alpha-amylase without sodium sulfite and acid detergent fiber (ADF) were determined as described by Mertens [11] and Licitra et al [12]. Lignin content was determined by treating the ADF residue with sulfuric acid at 72% [13]. Non-fiber carbohydrate (NFC) contents were calculated as described by Detmann et al [13]: NFC (g/kg) = 100–ash–EE–NDFap–CP; where ash is mineral matter (crude ash).

The total digestible nutrients (TDN) were estimated using the formula proposed by NRC [14]. To analyze the indigestible NDF, feed samples were placed in nonwoven fabric bags (20 mg/cm^2^) and incubated in the rumen for 288 hours ([13]; method INCT-CA F-008/1). Two adult crossbred cattle cannulated in the rumen and weighing 480±30 kg, with a mean age of 8 years were used for sample incubation. To determine the digestibility of each fraction, we used the following equation: ([ingested nutrient amount – amount nutrient excreted in the feces]×100) / ingested nutrient amount.

### Nitrogen balance and nitrogen concentration in the milk, blood, and urine

We collected samples of milk from each animal twice a day during the last five days of the trial period, and we measured the total amount of milk produced in the morning and afternoon. Fifty milliliters of the collected samples were added to a bottle containing the preservative Bronopol and then analyzed for milk urea nitrogen (MUN). The concentrations of MUN were determined by enzymatic and spectrophotometric methods of transreflectance using a ChemSpeck 150 (Uniontown, OH, USA).

Blood samples were collected from the coccygeal vein into vacuum tubes containing sodium fluoride and potassium oxalate (Glistab anticoagulant; Labtest Diagnóstica S.A., Lagoa Santa, Brazil) 4 hours after the morning feeding on the last day of the experimental period. The samples were centrifuged at 4,000 rpm for 20 min and the serum obtained was conditioned in Eppendorf tubes and frozen at −18°C for further analysis. Plasma urea concentrations were determined by a colorimetric enzymatic method using commercial kits (Ureia 500, Doles Reagents; Panamá, Brazil).

Urine spot samples were obtained during the experimental period, approximately four hours after feeding, during spontaneous urination. An aliquot of 10 mL of the urine sample was filtered and immediately diluted in 40 mL of H_2_SO_4_, 0.036 N for later analysis of urea and creatinine, as described by Oliveira et al [15]. The samples were then transferred to Eppendorf tubes and analyzed for urea content using the same method as was used for the blood samples. The end-point method determined the creatinine by means of picrate and acidifying with enzymatic methods. Quantification of the daily urinary volume of each animal was obtained by multiplying the respective BW by the amount of creatinine excreted daily, and then dividing the product by the creatinine concentration (mg/L) in the spot sample. The average value of 24.04 (mg/kg BW) was used, according to Chizzotti et al [16], to obtain the total daily creatinine excretion.

To perform the nitrogen balance calculation, the ingested amount of nitrogen (N-ingested; g/d) and the amount excreted in the feces (N-feces; g/d), urine (N-urine; g/d), and milk (N-milk; g/d) were used. The nitrogen utilization efficiency (NUE) of the diet was calculated by dividing the concentration of the nitrogen retained in the milk by the nitrogen intake in kg/d [17].

Feed efficiency was calculated by dividing the average milk yield (kg/d) by the DM intake (kg/d) [18]. The evaluation of costs with concentrates, roughage and total diet were calculated by multiplying the intake by the respective value of each fraction, which was calculated according to its composition and the price of each ingredient [19]. The values per kilogram of the diet ingredients were as follows: corn silage was $ 0.05 and concentrate was $ 0.46. The values are expressed in US dollars, considering the ratio of R$ 3.5 (real) for every US$ 1.

### Feeding behavior evaluations

The feeding behavior was assessed in the last 2 days of the trial period. For the evaluation of the feeding behavior, all animals were observed visually for 24 h, and the observations were recorded at 5-min intervals, which included eating, ruminating, and idle times [20]. On the same day, three observations were made for each animal: in the morning, at noontime, and at night. Data were collected by trained observers using digital timers. During the nocturnal observation, the environment was kept under artificial light. Feeding behavior variables (eating, ruminating, and idle times) were obtained by using equations adapted from Bürger et al [21]. The number of chews per ruminal bolus and the time spent ruminating each bolus were recorded during the observation periods. The number of bolus ruminated daily was calculated by dividing the total rumination time (min) by the average time spent to ruminate one bolus.

### Production, performance and body condition scores

During the trial period, the MP was recorded per cow. The MP was corrected for the fat content (FC) 3.5% using the equation proposed by Sklan et al [22]: MP 3.5% = MP×(0.432+ 0.163×FC).

To evaluate the BW of the animals, we used a mechanical scale. The animals were weighted at the beginning and end of the experiment. Body condition scores (BCS) were evaluated by a single technician weekly during the period. The BCS were also examined for three weeks further, following the end of the experimental period, to investigate the development of the animals. In the assessment of the BCS, the 1 to 5-point scale with 0.25-point intervals was used, in which 1 represents a very lean cow and 5 a very fat cow [23].

### Statistical analysis

The data was analyzed using the PROC MIXED procedure of SAS [24] (SAS Institute Inc., Cary, NC, USA). The model included treatment and period (time) as fixed effects. Results were reported as least squares means. Polynomial regressions were used to test the linear and quadratic changes affected due to the increasing feed restriction. Diagnostics concerning homogeneity of the variances and the normality of the residuals were examined and were not of concern for the variables studied here. Significant differences was declared at p<0.05.

## RESULTS

### Intake and digestibility of nutrient

For each 1% BW of feed restriction the DM intake was reduced by 5.26 kg/d (DMI, p<0.01), the CP by 0.61 kg/d (CPI, p<0.01), the NFC by1.82 kg/d (p<0.01), the neutral detergent fiber corrected for ash and protein by 2.43 kg/d (NDFap; p< 0.01) and the TDNs by 2.61 kg/d (p<0.01) (Table 2). The apparent digestibility of DM (p = 0.29), EE (p = 0.11), NFC (p = 0.49), NDFap (p = 0.11) and TDN (p = 0.39) was not differed with the feed restriction of the animals, with the averages being 59.20%, 82.35%, 76.87%, 54.50%, and 61.93%, respectively. There was a 19.06% increase in CP digestibility (p = 0.03) with feed restriction.

### Nitrogen balance and nitrogen concentration in the milk, blood, and urine

Ingested nitrogen (p<0.01) and nitrogen excreted in milk (p = 0.01) and feces (p<0.01) was linearly reduced with dietary restriction. There was a decrease of 45.76% in ingested nitrogen when the diet supply was limited from 3.39% to 2.00% of BW. Nitrogen losses in milk and feces were in the order of 19.57 and 61.20 g/d, respectively, per unit percentage reduction in supply. For nitrogen excreted in the urine, the means were adjusted to the quadratic regression model (p = 0.05), with the highest concentration occurring at the restriction level of 3.05% of BW supply (Table 3).The nitrogen balance (nitrogen retained) was reduced (p<0.01) by 36.39 g/d with the restriction in the diet supply of 3.39% to 2.0% of BW. It was verified that NUE was not altered with the restriction in the diet supply (mean 0.26; p = 0.59). The feed restriction of the cows did not modify the content of urea nitrogen present in the urine (p = 0.11) and milk (p = 0.17), with averages being 7.99 and 15.86 mg/dL, respectively. There was an increase of 3.66 mg/dL in the concentration of nitrogen in the plasma when the diet supply was reduced by 1%.

### Feeding behavior evaluations

Cows without restrictions in their diets spent 307.8 min/d more eating when compared to animals with a restriction of 2.00% BW (p<0.01; mean 132.6 min/d). There was a reduction of 225.7 min/d, 9.48 min/kg DM (p = 0.02) and 20.09 min/kg NDFap (p = 0.01) in the feeding time when restricted to 2.00% of BW (Table 4). For each 1% of feed restriction, there was a reduction of 107 min/d in rumination time. When expressed as min/kg DM (p = 0.26) and min/kg NDFap (p = 0.47), the rumination efficiency was not altered by the diet supply restriction, averaging 1.926 min/kg DM and 957.2 min/kg NDFap. The number of bolus chews (p = 0.55), as well as chewing time in min/kg DM (p = 0.62) and min/kg NDFap (p = 0.39) did not change due to feed restriction in lactating cows. The number of chews per day (p = 0.04) was reduced by 38.34% in the animals with a 2.0% restriction supply when compared to cows without restriction (26,706 chews/d). When expressed in min/d, the chewing time was reduced by 338.32 min for each 1% of diet restriction, and cows fed a 2.0% BW supply level spent less time (444 min/d) for this activity. The idle time was 45.42% higher in these animals than in animals without any diet restriction (mean of 543.6 min/d). The number of feeding periods (p<0.01) decreased by 76.92% for the restricted animals. For each percentage unit of diet supply restriction, there was a reduction of 7.17 feedings/d (Table 5). The duration of the feeding period (p = 0.35) and rumination (p = 0.35) was not influenced by feed restriction. An increase of 19.92 min for the duration of the idling period was observed in animals with a supply restriction of 2.0% BW. The feed efficiency of DM (p = 0.03) and NDFap (p = 0.02) in grams/h increased linearly with the restriction of the diet supply.

### Production, performance, and body condition scores

There was no difference in daily milk production (p = 0.09) with restriction in the diet supply to 3.39% to 2.0% of BW. When corrected for 3.5% fat, milk production decrease (p = 0.05) 3.46 kg/d for each percentage unit of feed restriction (Table 6). It was verified that for each 1% of feed restriction the final BW of the cows in the middle third of their lactation period was reduced by 46.75 kg, with the lowest values observed in animals with the highest level of restriction (2.0%, 438 kg). The final BW differential in relation to the initial weight was not influenced by restriction (p = 0.56), nor was the final BCS (p = 0.65). Dietary restriction did not affect feed efficiency (p = 0.49), with a mean of 0.93 kg of milk/kg of DMI. Feed restriction of 3.39% BW to 2.0% reduced diet costs of $ 3.25 to $ 1.77.

## DISCUSSION

The DMI is one of the main factors affecting animal health and performance [24]. According to the NRC, DMI is a function of the metabolic weight of the animal, week of lactation, and FC in milk and is estimated at 20.61 g/kg of BW (9.99 kg/d) for crossbred cows. However, a voluntary DMI of 32.74 g/kg of BW (15.88 kg/d) during the middle third of the lactation period is a greater value than what is estimated by the NRC [14] for maintenance and production. This difference can be explained by the fact that the NRC calculates these estimates based on Holstein cows in temperate regions. Murta et al [25] evaluated the productive performance of crossbred Holstein/Zebu cows from 100 to 150 days of lactation consuming different diets without restriction in the supply and verified that the DMI was about 33.0 g/kg of BW (15.7 kg/d). This value is similar to those that were observed in the present study (15.88 kg/d). Only in the restriction levels of 2.25% and 2.0% did the DMI drop below what was estimated by the NRC [14]. Consequently, restriction of the diet supply by up to 2% of an animal’s BW directly impacted the protein and energy intake of the animal, as well as the intake of other nutrients. However, the DM digestibility of the diet was not changed, with a mean of 592 g/kg DM. This occurred because the unique variation factor was the quantity offered. Normally in digestive trials, treatment of *ad libitum* (3.39% BW) feeding would reduce the digestibility rather than restricted feeding, because of less retention time in digestive tract comparing with low DMI [26]. However, this difference was only numerical in the order of 11.16% in *ad libitum* treatment (3.39% BW) with the restriction of 2% of BW.

The ingestive behavior and the speed of passage in the di gesta can modify the digestibility of nutrients because cows under restriction have more time for rumination compared to those without restriction, allowing for greater fractionation of the particles of the ruminal bolus, and favoring the degradation of the rumen microorganisms. However, this behavior related to the time spent ruminating the DM and the fibrous fraction was not altered with feed restriction, nor was the number of chews per bolus ruminated or the chewing time of the DM and NDFap affected.

The maintenance of these rumination characteristics through out the different diet supply levels can help to explain the similarity in DM digestibility and fibrous fraction values; this was because the diet was unique, with the same chemical composition across the different levels of supply and caused a longer feeding time in animals subjected to feed restriction [27,28]. The highest values observed for CP digestibility were in animals with restricted feed, which may be related to the rate of passage of the digesta functioning better due to the lower intake of DM in these animals. Lower digestion rates may imply a longer exposure of protein fractions to the action of microorganisms and proteolytic enzymes, which increases the digestibility of the CP in the rumen or intestine [28]. However, there are several isolated and interacting factors that influence the digestibility of nutrients in the gastrointestinal tract of animals [24].

Feed restriction in lactating cows reduces the ingested ni trogen and nitrogen balance due to the lower consumption of CP but does not modify the efficiency of nitrogen use (mean of 26%). Doska et al [29] reported that the nitrogen use efficiency can vary from 15% to 40% depending on the level of milk production and feeding practices. Plasma urea nitrogen was increased from 11.53 mg/dL (without restriction, 3.39% of BW) to 17.23 mg/dL (2.0% supply restriction). The increase in CP was not accompanied by an increase in the digestibility of the energy sources for the microorganisms of the rumen; with the quantitative restriction of food possibly hampering the use of rumen ammonia in the synthesis of microbial protein and increasing plasma urea nitrogen [28]. According to Doska et al [29], urea nitrogen and concentrations of nitrogen compounds in the plasma are related. In Brazilian conditions, values from 10.0 to 14.0 mg/dL of nitrogen in the plasma would represent the limits of dietary protein losses occurring. Thus, the restriction of dietary supply below 2.5% of DMI per BW increases the urea plasma concentration, which could indicate that there is a loss of protein occurring or an inefficient use of nutrients.

The reduction in the final BW of the cows with feed restric tion is explained by the lower intake of nutrients, however, the body score (mean 3.76) and feed efficiency (0.93 kg milk/kg of ingested DM) were maintained in these animals. This behavior demonstrates the adaptability potential of crossbred cows under conditions of diet supply restriction. Roche et al [30] evaluated strategies for increasing the BCS of pre- and postpartum cows and verified that the BCS is a metabolic indicator of the energy balance of the animal and can be used as a dietary energy restriction strategy. Roche et al [30] suggested that the ideal BCS for Holstein cows varies from 2.75 to 3.25 for cows in the middle third of their lactation periods, indicating that the crossbred cows used in this study had reserves slightly above those recommended for purebred cows.

It is important to emphasize that, in addition to unaltered milk production and maintenance BCS of cows with a restriction in diet supply, production costs declined by up to 45.62% in relation to the control diet (3.39% BW). This is very important since the profit margin within the dairy industry in Brazil is generally narrow. Moreover, the cost of the feed is greater than 50% of the total production cost. This strategy of restriction in the diet supply can be an alternative to make the production system feasible and to improve profitability. Therefore, the best restriction strategy can be evaluated within each milk production system. However, although there was no change in milk production or BCS in F1 Holstein/Zebu cows with diet restriction at 2% BW, there was a reduction in the milk production corrected for 3.5% fat and final BW of the animals, which could compromise reproductive activity. In this sense, dietary restriction of no less than 2.5% BW would be recommended in the middle third of lactation, in view of the lower weight loss of the cows and reduction of the food cost by as much as 28.42% compared to diet *ad libitum* (control; 3.25 US$/d).

## CONCLUSION

The restriction of diet to as low as 2% of an animal’s BW was effective in reducing food costs by 45.6%. This restriction of diet may be adopted due to the availability of food, milk prices, and other factors that interfere with the production system. However, considering the observed BW loss and decrease in milk production corrected for 3.5% fat, a restriction of no less than 2.5% BW is recommended.

## Figures and Tables

**Table 1:** Chemical composition of ingredients and diet used during experimental period

Items (g/kg DM)1)	Corn silage	Concentrated mixture	Diet2)
Dry matter	447.7	925.9	567.2
Organic matter	961.2	922.3	951.5
Crude protein	72.4	218.3	108.8
NDIN	5.50	12.24	7.18
ADIN	0.93	0.75	0.89
Ether extract	25.1	28.3	25.9
Non-fibrous carbohydrates	283.4	371.7	305.5
NDFap	580.3	304.1	511.3
Neutral detergente fiber	307.4	72.2	248.6
Lignin	60.3	31.8	53.2
Total digestible nutrients3)	606.4	734.2	638.4

DM, dry matter; NDIN, neutral detergent insoluble nitrogen; ADIN, acid detergent insoluble nitrogen; NDFap, neutral detergent fiber corrected for ash and protein.

1) Nutrient in dry basis (grams per kilogram).

2) Diet used during the experiment (e.g., diet consisted of 75% corn silage and 25% concentrate in the total dry matter).

3) NRC [14].

**Table 2:** Nutrient intake and digestibility in F1 Holstein/Zebu cows under quantitative feed restriction in the middle third of lactation

Items	Levels of restriction (% BW)1)					SEM	p-value	
	
	3.392)	2.75	2.50	2.25	2.00		Linear	Quad
Intake								
DM (kg/d) ^a)^	15.88	12.56	11.39	9.73	8.64	0.44	<0.01	0.90
DM (% BW)	3.22	2.75	2.50	2.25	2.00	0.06	<0.01	0.27
CP (kg/d) ^b)^	1.79	1.37	1.24	1.06	0.94	0.05	<0.01	0.68
EE (kg/d) ^c)^	0.42	0.33	0.29	0.25	0.22	0.01	<0.01	0.91
NFC (kg/d) ^d)^	5.14	3.84	3.47	2.97	2.63	0.15	<0.01	0.42
NDFCP (kg/d) ^e)^	7.77	6.42	5.83	4.98	4.42	0.22	<0.01	0.40
TDN (kg/d) ^f)^	9.12	7.63	7.21	6.18	5.47	0.29	<0.01	0.33
Nutrient digestibility (%)								
DM	54.75	57.38	61.62	60.65	61.63	2.59	0.29	0.76
CP ^g)^	51.88	48.15	59.25	55.46	61.77	2.63	0.03	0.12
EE	77.55	83.75	85.17	83.23	82.09	2.25	0.11	0.11
NFC	75.57	74.46	76.84	80.71	76.77	3.07	0.49	0.89
NDFAP	51.02	54.10	56.67	54.47	56.25	2.28	0.11	0.57
TDN	58.21	60.52	63.85	63.39	63.71	2.37	0.39	0.72

BW, body weight; SEM, standard error of the mean; p, probability; DM, dry matter; CP, crude protein; EE, ether extract; NFC, Non-fibrous carbohydrates; NDFap, neutral detergent fiber corrected for ash and protein; TDN, total digestible nutrients.

1) Regression equation: ^a)^ Ŷ = −1.93+5.26*X, R^2^ = 0.99; ^b)^ Ŷ = 0.27+0.88*X, R^2^ = 0.99; ^c)^ Ŷ = −0.30+0.61*X, R^2^ = 0.99; ^d)^ Ŷ = −0.07+0.14*X, R^2^ = 0.99; ^e)^ Ŷ = −1.08+1.82*X, R^2^ = 0.99; ^f)^ Ŷ = −0.394+2.43*X, R^2^ = 0.99; ^g)^ Ŷ = 0.388+2.61*X, R^2^ = 0.98, where X is the level of food restriction; R^2^ is the coefficient of determination; * significant by the t test, at 1% probability.

2) Diet *ad libitum*, allowing 5% refusals regarding dry matter offer.

**Table 3:** Balance and efficiency of nitrogen use in crossbred F1 Holstein/Zebu cows under quantitative feed restriction in the middle third of lactation

Items	Levels of restriction (% BW)1)					SEM	p-value	
	
	3.392)	2.75	2.50	2.25	2.00		Linear	Quad
N-ingested (g/d) ^a)^	298.13	218.50	211.15	171.04	161.70	12.86	<0.01	0.46
N-milk (g/d) ^b)^	69.13	56.72	55.72	48.89	40.24	4.47	0.01	0.52
N-feces (g/d) ^c)^	144.27	113.32	86.05	75.75	61.45	9.03	<0.01	0.86
N-urine (g/d)	58.55	50.35	72.39	67.80	82.82	7.36	0.05	0.13
Nitrogen balance ^d)^ (g/d)	26.17	−1.89	−3.00	−21.40	−22.81	7.62	<0.01	0.75
NUE^e)^	0.23	0.26	0.27	0.29	0.25	0.02	0.59	0.36
UUN (mg/dL) ^f)^	7.46	10.92	9.95	4.88	6.75	1.58	0.11	0.34
PUN (mg/dL)	11.53	14.20	13.56	15.10	17.23	1.03	0.02	0.48
MUN (mg/dL)	15.15	13.70	16.63	15.75	18.10	1.21	0.17	0.16

BW, body weight; SEM, standard error of the mean; p, probability; N, nitrogen; NUE, nitrogen use efficiency; UUN, urine urea nitrogen; PUN, plasma urea nitrogen; MUN, milk urea nitrogen.

1) Regression equation: ^a)^ Ŷ = −45.61+99.96*X, R^2^ = 0.97; ^b)^ Ŷ = 3.67+19.57*X, R^2^ = 0.96; ^c)^ Ŷ = −61.61+61.20*X; R^2^ = 0.98; ^d)^ Ŷ = 289.80–153.66*X+25.13*X^2^, R^2^ = 0.76; ^e)^ Ŷ = −98.41+36.39*X, R^2^ = 0.96; ^f)^ Ŷ = 23.77–3.66*X, R^2^ = 0.87 where X is the level of food restriction; R^2^ is the coefficient of determination; * significant by the t test, at 1% probability.

2) Diet *ad libitum*, allowing 5% refusals regarding dry matter offer.

**Table 4:** Feeding behavior of F1 Holstein/Zebu cows under quantitative feed restriction in the middle third of lactation

Items	Levels of restriction (% BW)1)					SEM	p-value	
	
	3.392)	2.75	2.50	2.25	2.00		Linear	Quad
Feeding								
min/d ^a)^	440.4	240	204	153.6	132.6	28.2	<0.01	0.09
min/kg DM^b)^	27.37	19.17	16.87	15.62	14.36	2.64	0.02	0.61
min/kg NDFap^c)^	55.69	37.5	33	30.56	28.08	5.16	0.01	0.32
Rumination								
min/d ^d)^	456.6	415.2	367.8	339	311.4	28.8	0.02	0.60
min/kg DM	2,219	1,828	1,980	1,785	1,818	149	0.26	0.60
min/kg NDFap	1,087	935	1,013	912	929	76	0.47	0.73
Chewing								
number/bolus	49.86	38.31	49.39	39.19	42.46	6.22	0.55	0.66
number/d ^e)^	26,706	21,034	20,191	18,181	16,466	2,117	0.04	0.81
min/d ^f)^	896.4	655.2	571.2	492.6	444	30	<0.01	0.24
min/kg DM	54.71	52.67	47.46	50.62	48.05	3.74	0.62	0.86
min/kg NDFap	111.5	103	92.8	99	94	7.2	0.39	0.65
Idleness								
min/d ^g)^	543.6	784.8	868.8	947.4	996	30	<0.01	0.26

BW, body weight; SEM, standard error of the mean; p, probability; DM, dry matter; NDFap, neutral detergent fiber corrected for ashes and protein.

1) Regression equation: ^a)^ Ŷ = −346.37+225.17*X, R^2^ = 0.96; ^b)^ Ŷ = −5.78+9.48*X, R^2^ = 0.95; ^c)^ Ŷ = −14.83+20.09*X, R^2^ = 0.94; ^d)^ Ŷ = 100.61+107*X, R^2^ = 0.97; ^e)^ Ŷ = −1809.76+7256.01*X, R^2^ = 0.99; ^f)^ Ŷ = −245+332.38*X, R^2^ = 0.99; ^g)^ Ŷ = 1685–332.38*X, R^2^ = 0.99 where X is the level of food restriction; R^2^ is the coefficient of determination; * significant by the t test, at 1% probability.

2) Diet *ad libitum*, allowing 5% refusals regarding dry matter offer.

**Table 5:** Number of periods and average time spent per period on the feeding, ruminating, and idle activities by F1 Holstein/Zebu cows under quantitative feed restriction in the middle third of lactation

Items	Levels of restriction (% BW)1)					SEM	p-value	
	
	3.392)	2.75	2.50	2.25	2.00		Linear	Quad
Number of periods (n/d)								
Feeding^a)^	13.0	4.5	4.7	3.2	3.0	0.71	<0.01	0.01
Ruminating	14.5	12.75	12	13.5	12.75	1.19	0.65	0.42
Idling	21	20.25	19.75	19.75	18.75	1.37	0.83	0.84
Time spent per period (min)								
Feeding	33.93	54.88	42.44	53.65	46.15	7.81	0.35	0.26
Ruminating	31.96	33.09	34.47	25.04	24.54	4.25	0.35	0.21
Idling^b)^	26	39.14	45.64	47.96	53.98	3.4	<0.01	0.81
Feed efficiency								
g DM/h^c)^	2,361	3,210	3,804	3,912	4,239	381	0.03	0.80
g NDFap/h^d)^	1,154	1,641	1,945	2,000	2,167	193	0.02	0.71
Rumination efficiency								
Boluses/d	49.86	38.31	49.39	39.19	42.46	6.22	0.55	0.70
g DM/h	2,219	1,828	1,980	1,785	1,818	149	0.26	0.48
g NDFap/h	1,087	935	1,013	912	929	76	0.47	0.66

BW, body weight; SEM, standard error of the mean; p, probability; DM, dry matter; NDFap, neutral detergent fiber corrected for ashes and protein; h, hour.

1) Regression equation: ^a)^ Ŷ = −12.80+7.17*X, R^2^ = 0.85; ^b)^ Ŷ = 93.90–19.92*X, R^2^ = 0.99; ^c)^ Ŷ = 7,044.02–1,372.74*X, R^2^ = 0.98; ^d)^ Ŷ = 3687.88–739.48*X, R^2^ = 0.98; where X is the level of food restriction; R^2^ is the coefficient of determination; * significant by the t test, at 1% probability.

2) Diet *ad libitum*, allowing 5% refusals regarding dry matter offer.

**Table 6:** Performance and feed efficiency in F1 Holstein/Zebu cows under quantitative feed restriction in the middle third of lactation

Items	Levels of restriction (BW)1)					SEM	p-value	
	
	3.392)	2.75	2.50	2.25	2.00		Linear	Quad
Milk production (kg/d)	13.05	11.60	10.70	10.67	8.26	1.10	0.09	0.47
Milk production corrected for 3.5 fat (kg/d)^a)^	13.61	11.30	11.30	10.27	8.32	1.10	0.05	0.59
Final body weight (kg) ^b)^	485	457	455	433	438	80	<0.01	0.57
Final and initial body weight difference (kg)	−24.25	−25.04	−56.31	−52.92	−69.94	23.04	0.56	0.66
Final body condition score	3.81	3.81	3.88	3.69	3.63	0.13	0.65	0.34
Difference in body condition score	0.06	0.00	−0.13	−0.25	0.00	0.09	0.16	0.29
Feed efficiency (kg of milk/kg of DM)	0.79	0.95	0.90	1.10	0.91	0.12	0.49	0.50
Feed cost (U$/d)	3.25	2.57	2.33	1.99	1.77	0.26	-	-
Reduction of food costs (%)	0.00	20.90	28.42	38.87	45.62	7.7	-	-

BW, body weight; SEM, standard error of the mean; p-value, probability; DM, dry matter.

1) ^a)^ Ŷ = 2.03+3.46*X, R^2^ = 0.93; ^b)^ Ŷ =356.4 – 46.75*X, R^2^ = 0.97, where X is the level of food restriction; R^2^ is the coefficient of determination; * significant by the t test, at 1% probability.

2) Diet *ad libitum*, allowing 5% refusals regarding dry matter offer.
